# Description and Phylogeny of *Tetrakeronopsis silvanetoi* gen. nov., sp. nov. (Hypotricha, Pseudokeronopsidae), a New Benthic Marine Ciliate from Brazil

**DOI:** 10.1371/journal.pone.0088954

**Published:** 2014-02-24

**Authors:** Thiago da Silva Paiva, Amanda Ferreira Cavalcante de Albuquerque, Bárbara do Nascimento Borges, Maria Lúcia Harada

**Affiliations:** 1 Laboratório de Protistologia, Departamento de Zoologia, Instituto de Biologia, Universidade Federal do Rio de Janeiro, Rio de Janeiro, RJ, Brazil; 2 Laboratório de Biologia Molecular “Francisco Mauro Salzano”, Instituto de Ciências Biológicas, Universidade Federal do Pará, Belém, PA, Brazil; 3 Universidade do Grande Rio, Duque de Caxias, RJ, Brazil; 4 Centro de Tecnologia Agropecuária, Instituto Socioambiental e dos Recursos Hídricos, Universidade Federal Rural da Amazônia, Belém, PA, Brazil; J. Craig Venter Institute, United States of America

## Abstract

Pseudokeronopsidae Borror & Wicklow, 1983 are biotechnologically important ciliate protists which produce toxic defense substances; however, their diversity is still little known in Brazil. In the present study, *Tetrakeronopsis silvanetoi*, a new genus and species of marine pseudokeronopsid hypotrichs is described from samples of water with bottom sediment collected from the coast of São Paulo state. Its phylogenetic affinities to the “core urostyloids” are hypothesized based on analyses of the 18S-rDNA marker, and a new subfamily, the Nothoholostichinae subfam. nov., is erected to name the monophylum composed of pseudokeronopsids in which the anterior corona is usually formed by four frontal cirri. In addition, the new combination *Monocoronella longissima* comb. nov. is proposed for *Nothoholosticha longissima* (Dragesco & Dragesco-Kernéis, 1986) Li *et al.*, 2009.

## Introduction

Hypotrichs *s. l.* [1] are ciliates which generally exhibit a dorsoventrally flattened body with a polyhymenophore adoral zone and somatic cilia arranged in cirri along the ventral side and rows of dikinetids on the dorsal side of the body [2]–[4]. Their body architecture makes them pre-adapted to life in micropore habitats [5] and, like most ciliates, hypotrichs occur in edaphic, freshwater and marine sediments worldwide [6]. In marine environments, hypotrichs are present mostly as free-living microbenthic forms, which are omnivorous and compete for food with similar sized interstitial metazoa and other protists [7], [8].

Among hypotrichs, representatives of the Pseudokeronopsidae are known to produce toxic substances, the keronopsins, which are used as chemical defense [9]–[12]. Hence, pseudokeronopsids offer biotechnological potential for drug discovery related studies, which makes expanding knowledge of their diversity highly desirable.

In Brazil, the most relevant studies on the diversity of benthic marine ciliates were those conducted by Kattar [13], who found 75 species along the coast of the states of São Paulo, Guanabara (nowdays part of Rio de Janeiro), Espírito Santo and Pernambuco; and by Wanick & Silva-Neto [14], who reported 32 species from Sepetiba bay, Rio de Janeiro. However, the diversity of benthic marine ciliates remains unknown for the majority of the Brazilian coast [15], and this includes the diversity of marine pseudokeronopsids.

The present study expands the knowledge on diversity of marine pseudokeronopsids from Brazil with the description of a new genus and species, namely *Tetrakeronopsis silvanetoi* gen. nov., sp. nov., found in environmental samples from the coast of São Paulo, Brazil. The systematics of the new organism is discussed based on comparative morphology and phylogenetic analyses of the 18S-rDNA marker, and a new subfamily, Nothoholostichinae subfam. nov., is proposed.

## Materials and Methods

### Morphology

Samples with water and bottom sediment were collected from Martim de Sá beach, in Caraguatatuba, a city on the coast of São Paulo state, Brazil, in May of 2006. The sampling location is a public beach, thus no specific permissions were required to collect the material necessary for the present study. No known endangered or protected species were involved in the present study.

The samples were brought to the laboratory, where aliquots were examined in Petri dishes under the stereomicroscope. For identification and description, the ciliates were further analyzed in vivo under bright field and DIC at 100×, 200× and 1,000× magnifications, and after protargol-impregnation and scanning electron microscopy, prepared according to Dieckmann [16] and Silva-Neto *et al.* [17], respectively. Measurements in Table 1 were taken from protargol-impregnated specimens at 1,000× magnification, and descriptive statistics therein were calculated with the computer program GraphPad Prism 4 [18]. All measurements are in µm. Diagrams illustrating the ciliature pattern are schematic representations drawn with Adobe PhotoShop CS4, and were based on photographs of various protargol-impregnated specimens. The terminology adopted in the present study follows mostly Berger [2], [19], and the classification shown in the phylogenetic tree is basically according to Chen *et al.* [20], except for *Anteholosticha* spp. (see discussion below).

**Table 1:** Morphometric characterization of *Tetrakeronopsis silvanetoi* gen. nov., sp. nov.

Character	Mean	M	SD	SE	CV(%)	Min	Max	N
Body length	236.4	234.5	38.1	8.1	16.1	178.0	305.0	22
Body width	37.2	35.0	5.0	1.1	13.4	26.0	45.0	22
Length of AZM	64.5	64.0	8.7	1.9	13.5	51.0	85.0	22
Number of AM in the crown	13.5	13.0	1.5	0.3	10.9	12	17	21
Number of AM in the lapel	43.0	43.0	4.2	0.9	9.8	40	52	21
Total number of AM	56.7	57.5	5.4	1.2	9.5	49	67	20
Distance from anterior end ofthe body to distal end AZM	11.6	11.0	1.5	0.3	12.7	10.0	15.0	22
Length of undulatingmembranes figure	26.8	26.0	5.8	1.4	21.6	19.0	35.0	17
Distance from anterior end ofbody to undulatingmembranes	36.0	35.0	5.3	1.2	14.6	30.0	45.0	19
Length of infundibular fibers	42.3	41.5	6.6	1.9	15.6	30.0	54.0	12
Number of frontal cirri	4.0	4.0	0	0	0	4	4	22
Number of parabuccal cirri	2.0	2.0	0	0	0	2	2	20
Number of frontoterminal cirri	2.0	2.0	0	0	0	2	2	20
Number of buccal cirri	1.1	1.0	0.4	0.1	31.1	1	2	15
Number of midventralcirri pairs	36.6	35.0	5.3	1.4	14.5	29	46	15
Distance from anterior end of body to LMR	25.8	25.5	2.4	0.5	9.4	20.0	30.0	22
Number of cirri in LMR	69.6	68.0	12.0	2.7	17.2	55	98	19
Distance from anterior end ofbody to RMR	19.8	20.0	1.6	0.4	8.1	16.0	23.0	20
Number of cirri in RMR	72.8	73.0	10.2	2.4	14.0	54	90	18
Number of pretransverse cirri	2.0	2.0	0	0	0	2	2	22
Number of transverse cirri	5.4	5.0	0.6	0.1	10.9	4	6	22
Number of dikinetids in DK1	38.0	41.0	6.8	3.0	17.9	27	43	5
Number of dikinetids in DK2	33.0	31.0	4.3	1.6	13.1	29	39	7
Number of dikinetids in DK3	33.3	35.0	3.1	1.2	9.3	29	36	7
Number of macronuclear nodules	37.5	37.0	4.7	1.0	12.4	30	47	22
Length of macronuclearnodules	9.5	10.0	2.6	0.5	26.9	5.0	13.0	22
Width of macronuclearnodules	4.5	4.5	1.1	0.2	23.5	2.0	6.0	22
Number of micronuclei	4.9	5.0	1.3	0.3	26.8	3	8	15
Length of micronuclei	4.7	5.0	0.6	0.2	12.5	4.0	6.0	15
Width of micronuclei	4.0	4.0	0.4	0.1	9.5	3.0	5.0	15

Legend: AM – adoral membranelles; AZM – adoral zone (of membranelles); CV – coefficient of variation; DK1– left dorsal kinety; DK2– middle dorsal kinety; DK3– right dorsal kinety; LMR – left marginal cirral row; M – median; Max – maximum value observed; Mean – arithmetic mean; Min – minimum value observed; N – sample size; RMR – right marginal cirral row; SD – standard deviation; SE – standard error.

### Phylogenetic Analyses

To assure correct identification of the ciliates used in the molecular analyses, clonal cultures were made from single specimens picked from ordinary cultures and transferred to Petri dishes with boiled sea water and crushed rice grains. Specimens were then isolated for DNA extraction and amplification of the 18S marker following Paiva *et al.* [21]. The obtained sequence was added to a data matrix containing 54 other sequences representing the “core urostyloids” [22]–[24], which is the largest monophylum of urostyloids recovered in molecular phylogenies, but also the group in which pseudokeronopsids are included, e.g. [20]–[24], [26]. Seven additional sequences of representatives of Discocephalida Wicklow, 1982, sensu [27] were included as an outgroup [25]–[27]. The sequences were aligned based on their eukaryotic 18S-rRNA secondary structure, using the SINA web aligner (http://www.arb-silva.de/aligner) [28] with its default settings. Next, the nucleotide matrix was inspected in the computer program BioEdit v7.0.6 [29] and their alignment refined by eye, considering the structural similarity among sequences. Genetic distances (Table 2) were calculated with the program MEGA 5 [30], using pairwise deletion as treatment for gaps and missing data. The nucleotide sequence of *Tetrakeronopsis silvanetoi* obtained in the present study was deposited in NCBI/GenBank (access code: KF730314).

**Table 2:** Distance matrix of 18S-rDNA sequences of representatives of the Pseudokeronopsidae.

	Sequences withaccession codes	1	2	3	4	5	6	7	8	9	10	11	12	13	14
	**Pseudokeronopsinae**														
**1**	AY881633 *P. carnea*														
**2**	JN714476 *P.carnea*	0.001													
**3**	FJ775723 *P. erythrina*	0.020	0.021												
**4**	AY881634 *P. flava*	0.019	0.019	0.012											
**5**	HM140386 *P. flava*	0.016	0.016	0.009	0.004										
**6**	DQ640314 *P. rubra*	0.018	0.019	0.009	0.008	0.005									
**7**	EF535729 *P. rubra*	0.031	0.031	0.021	0.021	0.017	0.013								
**8**	HM140387 *P. rubra*	0.016	0.017	0.006	0.006	0.003	0.003	0.015							
**9**	FJ870094 *U. citrina*	0.020	0.021	0.015	0.010	0.008	0.010	0.022	0.008						
**10**	GU437211 *U. citrina*	0.022	0.021	0.016	0.011	0.010	0.011	0.022	0.010	0.005					
**11**	JN714477 *U. citrina*	0.022	0.021	0.016	0.011	0.009	0.010	0.022	0.010	0.005	0.001				
	**Nothoholostichinae** **subfam. nov.**														
**12**	JQ955541 *A. sinica*	0.036	0.036	0.036	0.045	0.042	0.043	0.046	0.042	0.044	0.043	0.044			
**13**	JQ083600 *H. pulchra*	0.039	0.040	0.042	0.047	0.045	0.046	0.056	0.045	0.048	0.048	0.048	0.019		
**14**	FJ377548 *N. fasciola*	0.040	0.041	0.044	0.046	0.045	0.046	0.059	0.045	0.050	0.051	0.051	0.017	0.017	
**15**	KF730314 *T. silvanetoi* gen.nov., sp. nov.	0.039	0.039	0.050	0.051	0.048	0.049	0.049	0.047	0.054	0.054	0.055	0.025	0.026	0.025

Bayesian inference (BI) and maximum likelihood (ML) analyses were performed to hypothesize the phylogenetic affinities of *T. silvanetoi* within the studied taxa sample. Both analyses employed the TrN+I ( = 0.4687)+Γ ( = 0.4828) nucleotide substitution model, selected via the Akaike information criterion (AIC) [31], [32] in MODELTEST 3.7 [33]. The BI was performed with the program MrBayes 3.2.1 implemented in the CIPRES Science Gateway (http://www.phylo.org) [34]. It was based on two independent Markov Chain Monte Carlo (MCMC) simulations run with four chains of 1,000,000 generations, and trees sampled each 200 (temperature of heat chains = 0.2). The first 100,000 generations were discarded as burn-in. For ML, the sequences were analyzed with the program PhyML 3.0 [35] using an initial BioNJ topology, and improving its likelihood via subtree pruning and regrafting (SPR) branch-swapping moves to achieve the ML tree. Node stability in BI was assessed via posterior probabilities calculated from a 50% majority-rule consensus of the trees kept after burn-in, and in ML via 1,000 bootstrap pseudoreplicates. The trees were rooted *a posteriori* according to outgroup position [36].

In the present study, we did not assess the phylogeny of the Urostyloidea Bütschli, 1889 as a whole, which are generally hypothesized as non-monophyletic, e.g. [21], [24], [26], [37]–[39], but rather focused our analyses on the core urostyloids.

### Nomenclatural Acts

The electronic edition of this article conforms to the requirements of the amended International Code of Zoological Nomenclature, and hence the new names contained herein are available under that Code from the electronic edition of this article. This published work and the nomenclatural acts it contains have been registered in ZooBank, the online registration system for the ICZN. The ZooBank LSIDs (Life Science Identifiers) can be resolved and the associated information viewed through any standard web browser by appending the LSID to the prefix “http://zoobank.org/”. The LSID for this publication is: urn:lsid:zoobank.org:pub: E5464A1D-5EA0-45A3-8D1E-26230E691A8C. The electronic edition of this work was published in a journal with an ISSN, and has been archived and is available from the following digital repositories: PubMed Central, LOCKSS.

## Results

Spirotrichea Bütschli, 1889

Hypotricha Stein, 1859 ( = Stichotrichia Small & Lynn, 1985)

Urostyloidea Bütschli, 1889

Pseudokeronopsidae Borror & Wicklow, 1983

Nothoholostichinae subfam. nov. urn:lsid:zoobank.org:act: 6F01D1BC-9961-4BE6-A630-4A15D957FE08

Name-bearing type genus: *Nothoholosticha* Li *et al*., 2009.

### Diagnosis

Pseudokeronopsidae sensu Chen *et al.* [20] with an atypical bicorona in which the anterior corona is usually formed by four frontal cirri.

### Genera Included

Nothoholosticha Li et al., 2009; *Apoholosticha* Fan *et al.*, 2013; *Heterokeronopsis* Pan *et al.*, 2013; and *Tetrakeronopsis* gen. nov (Table 3).

**Table 3:** Morphological comparison among genera of the Nothoholostichinae subfam. nov.

Character	*Apoholosticha*	*Heterokeronopsis*	*Nothoholosticha*	*Tetrakeronopsis* gen. nov.
Frontoterminal cirri, presenceand number	present, four	absent	absent	present, two
Buccal cirri, presence	absent	present	present	present
Posterior midventral cirral rowformed by rightmost ventralprimordium, presence	absent	present	absent	absent
Distinct pretransverse cirri,presence	absent	absent	absent (?)	present
Transverse cirri, presence	present	absent	present	present
Data source	[23]	[40]	[41]	This study

*Tetrakeronopsis* gen. nov. urn:lsid:zoobank.org:act: 4770CAC8-4586-4DBD-AC70-178D803E16E7.

### Etymology

Greek, composite of prefix *tetra*- (four) and *Keronopsis* Penard, 1922. Named after the number of cirri composing the anterior corona; feminine.

### Diagnosis

Nothoholostichinae with two frontoterminal cirri; buccal, pretransverse and transverse cirri present; midventral complex formed by cirral pairs only; one left and one right marginal cirral row; caudal cirri absent.

Type species: *Tetrakeronopsis silvanetoi* sp. nov.

### Species Included

Up to the present time, only the type species *T. silvanetoi* is assigned to *Tetrakeronopsis.*

*Tetrakeronopsis silvanetoi* sp. nov. urn:lsid:zoobank.org:act: 76EFD829-3679-42CE-B61C-56869A060ABC.

### Etymology

T. da S. Paiva proposed the epithet “*silvanetoi*” in dedication to his former doctoral advisor Prof. Dr. Inácio Domingos da Silva Neto, who collected the environmental samples in which this new species was found.

### Diagnosis

*Tetrakeronopsis* measuring ∼ 280×40 µm in vivo (N = 15); body slender, flexible and acontractile, of pale yellow coloration under the stereomicroscope, exhibiting a conspicuous longitudinal ventral groove; rusty colored cortical granules present but scarce; cytoplasm with many globular and ring-shaped inclusions. Adoral zone with crown and lapel membranelles separated by a gap; right portion of lapel membranelles separated by a gap; on average 14 membranelles in the crown and 43 in the lapel; two parabuccal and one (rarely two) buccal cirri; midventral complex formed by 37 cirral pairs; 70 left and 73 right marginal cirri; two pretransverse and 5 transverse cirri; three dorsal kineties. Nuclear apparatus with, on average, 38 macronuclear nodules and five micronuclei.

### Type Locality

The sand beach of Martim de Sá, in Caraguatatuba city, São Paulo state, Brazil. Geographic coordinates: −23.626911, −45.380879.

### Deposition of Type-specimens

Type slides (protargol-impregnation) of *T. silvanetoi* were deposited in the collection of Laboratório de Protistologia, Dept. de Zoologia, Inst. de Biologia, Universidade Federal do Rio de Janeiro, under the accession codes IBZ-UFRJ0014-3– holotype (marked with ink on the slide) and various paratypes; and IBZ-UFRJ0014-4– paratypes.

### Morphology

#### Interphase

**Figure 1:**
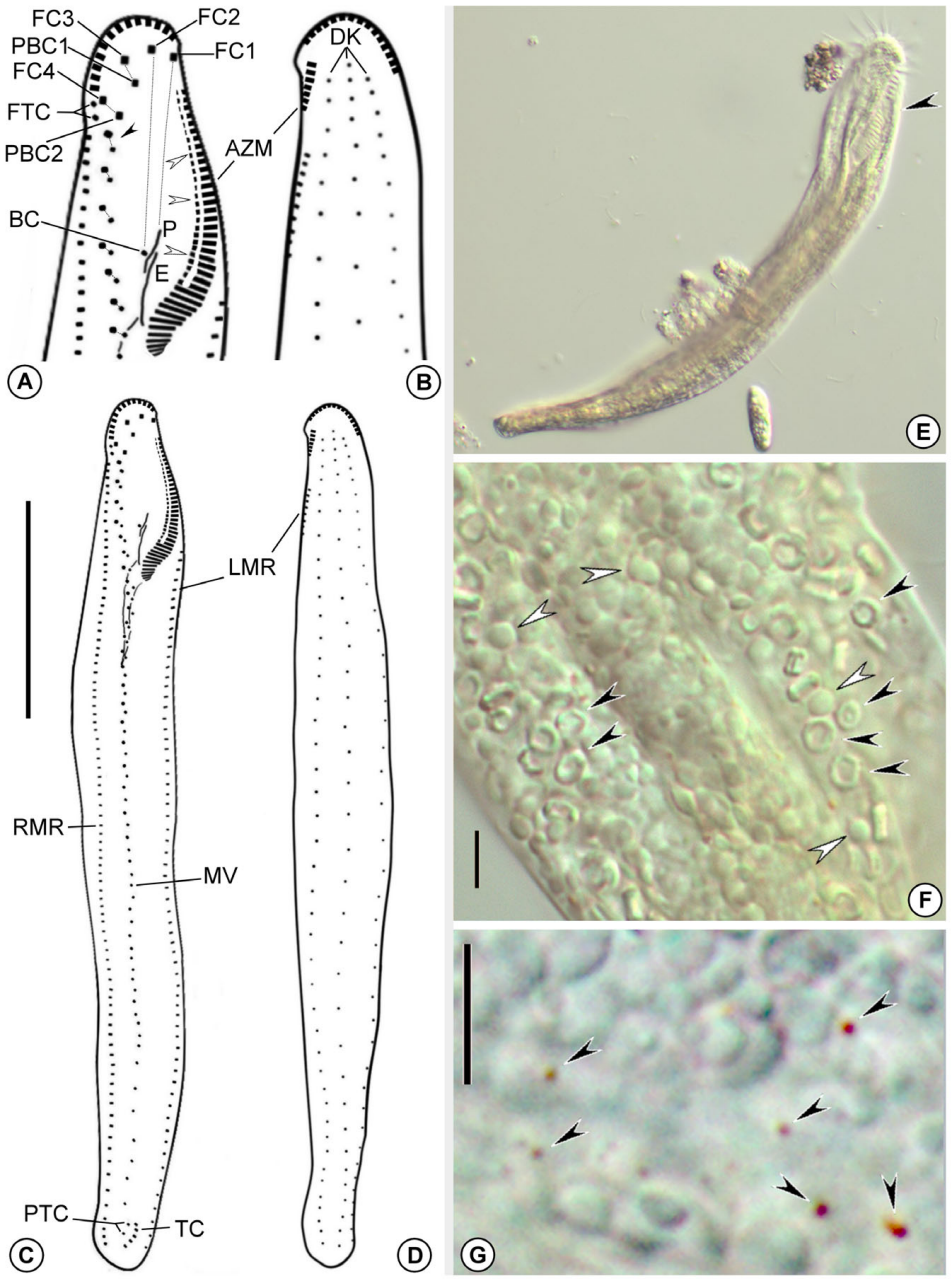
*Tetrakeronopsis silvanetoi*. **A–D.** Schematic diagrams representing the ciliature organization of *T. silvanetoi* after protargol-impregnation. **A.** Detail of anterior region of body showing frontal and oral ciliatures. The black arrowhead points to anteriormost midventral cirral pair, and the white arrowheads show a gap in the lapel adoral membranelles; **B.** Detail of dorsal side showing anterior dorsal ciliature; **C.** Ventral ciliature; **D.** Dorsal ciliature; **E–G.** Live organism. **E.** Specimen showing habitus. Arrowhead points to adoral zone; **F.** Cytoplasmic inclusions. Black arrowheads mark ring-shaped bodies; white arrowheads mark globular bodies. **G**. Detail of cortical granules (arrowheads). Legend: AZM – adoral zone (of membranelles); BC – buccal cirrus; DK – dorsal kineties; E – endoral; FC(n) – frontal cirrus; FTC – frontoterminal cirri; PBC(n) – parabuccal cirrus; LMR – left marginal cirral row; MV – midventral complex; P – paroral; PTC – pretransverse cirri; RMR – right marginal cirral row; TC – transverse cirri. Scale bars: **C–D**. 60 µm; **F–G**. 4 µm.

Body slender and rather flexible, but mostly acontractile. Moves moderately slowly on bottom of Petri dishes, crawling among debris, without thigmotactic behavior. Specimens pale yellow under stereomicroscope. Cytoplasm clear transparent under high magnification, filled with numerous transparent ring-like bodies ∼2 µm in diameter, characteristic of pseudokeronopsids, and variable sized (∼1–2 µm) globules. Cortical granules inconspicuous (≤0.5 µm), rusty colored, scarce and scattered (Figure 1A–C). Contractile vacuole not found, thus assumed to be lacking; food vacuoles containing mostly bacteria.

**Figure 2:**
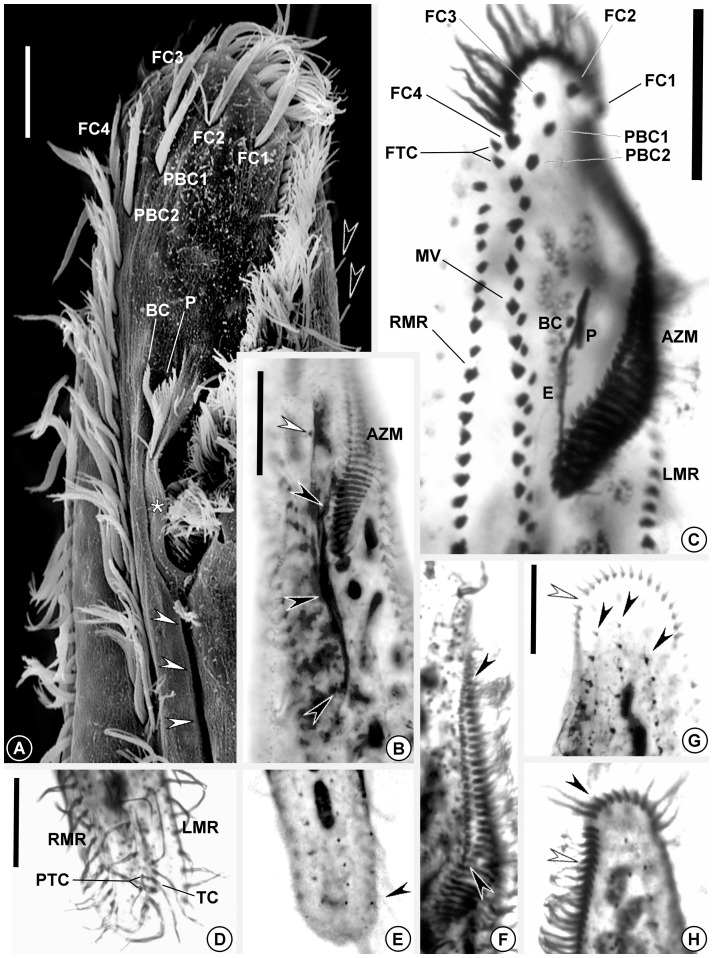
Electron and optical micrographs of *Tetrakeronopsis silvanetoi*. **A.** Scanning electron microscopy image showing aspects of anterior region of body. Asterisk marks peristomal lip; black arrowheads point to dorsal bristles; white arrowheads show longitudinal ventral groove; **B–H.** Specimens after protargol-impregnation. **B.** Ventral side of specimen showing detail of infundibular fibers (black arrowheads). The white arrowhead shows buccal cirrus; **C.** Detail of anterior region of body after protargol-impregnation; **D.** Posterior ventral region, showing transverse ciliature; **E.** Posterior termini of dorsal kineties. The arrowhead indicates the terminus of right dorsal kinety; **F.** Detail of adoral zone showing gaps in lapel membranelles (arrowheads); **G.** Detail of anterior region of body, dorsal side. The black arrowheads show dorsal kineties, and the white arrowhead indicates the gap which splits the adoral zone; **H.** Detail of dorsolaterally placed adoral membranelles in the distal region of lapel (white arrowhead) and the crown adoral membranelles (black arrowhead). Legend: AZM – adoral zone (of membranelles); BC – buccal cirrus; E – endoral; FC(n) – frontal cirrus; FTC – frontoterminal cirri; PBC(n) – parabuccal cirrus; LMR – left marginal cirral row; MV – midventral complex; P – paroral; PTC – pretransverse cirri; RMR – right marginal cirral row; TC – transverse cirri. Scale bars: **A.** 10 µm; **B–H.** 20 µm.

Adoral zone occupying ∼ 22% of body length in vivo (N = 15) (∼ 27% after protargol impregnation), with a DE-value [2] of 0.18. Adoral zone split by a ∼ 2.5 µm wide gap, thus crown and lapel regions distinctly separated (Figures 1A–E; 2A–C, G–H). Crown forming an arch of 12–17 membranelles with ∼15–16 µm long cilia; lapel slightly dorsolateral at its distal portion, arranged in a more-or-less gonostomoid pattern and having 40–52 membranelles, in which the longest cilia measure ∼ 11 µm. Except for the ∼ 15 proximal ones, membranelles of the lapel have their right portion separated by a gap (missing basal bodies?) (Figures 1A; 2A, C, F). Undulating membranes almost straight, not optically intersecting each other (Figures 1A; 2A, C), measuring 19–35 µm long and placed at 30–45 µm from anterior end of body; paroral remarkably shorter than endoral. Paroral cilia about 5 µm long and attached on a short peristomal lip which borders a narrow oral cavity (Figure 2A). Behind peristomal lip, a conspicuous longitudinal ventral groove, easily seen even in live specimens at low magnification, extends to near posterior region of midventral complex (Figure 2A). Infundibular fibers 30–54 µm long (Figure 2B).

Frontal ciliature arranged in an atypical bicorona invariably formed by four frontal and two parabuccal cirri, each ∼ 13 µm long (Figures 1A; 2A, C). Leftmost frontal cirrus (FC1) located near gap between crown and lapel; rightmost frontal cirrus (FC4) immediately behind distal end of adoral zone. Invariably two frontoterminal cirri right of FC4. One (rarely two) buccal cirrus adjacent to the right of paroral (Figures 1A; 2A–C).

Midventral complex formed by 29–46 cirral pairs, running in about the median of the body on the ventral surface, between right marginal cirral row and the above mentioned ventral groove, terminating near pretransverse cirri (Figures 1A, C; 2A, C–D). Midventral pairs slightly oblique above body equator, displaying a typical urostyloid zig-zag pattern; below the equatorial region, midventral pairs become less oblique, almost aligned as a straight row. Pairs located close to transverse cirri are more spaced than in the rest of the complex. Cirri of midventral complex ∼ 12 µm long, with the right cirrus of each pair slightly thicker than the left one (Figure 2C). Two thin pretransverse and an almost longitudinal set of 4–6 about 17 µm long transverse cirri in the posterior region of the body.

Marginal ciliature composed of one left and one right cirral rows bearing ∼ 11–12 µm long cilia. Left marginal row with 55–98 cirri, beginning dorsally, at 20–30 µm from anterior end of body, shifting to ventral side and running along left margin, terminating close to posterior end of body; right marginal row with 54–90 cirri, beginning at ∼ 16–23 µm away from anterior end of body, at the level of anteriormost midventral cirrus, running along right margin and terminating near posterior end of body, at the level of left marginal row terminus. Both rows straight, ending parallel at, on average, 8.4 µm (N = 6) from posterior end of body and separated from each other by a ∼ 13 µm wide gap (Figures 1A–D; 2C–D).

Dorsal ciliature composed of three rows of ∼ 3 µm long bristles; middle row always beginning slightly anteriorly in relation to the other two; all three rows terminate at about the same level, on average 8.3 µm (N = 6) from posterior end of body; caudal cirri lacking (Figures 1D; 2E, G).

**Figure 3:**
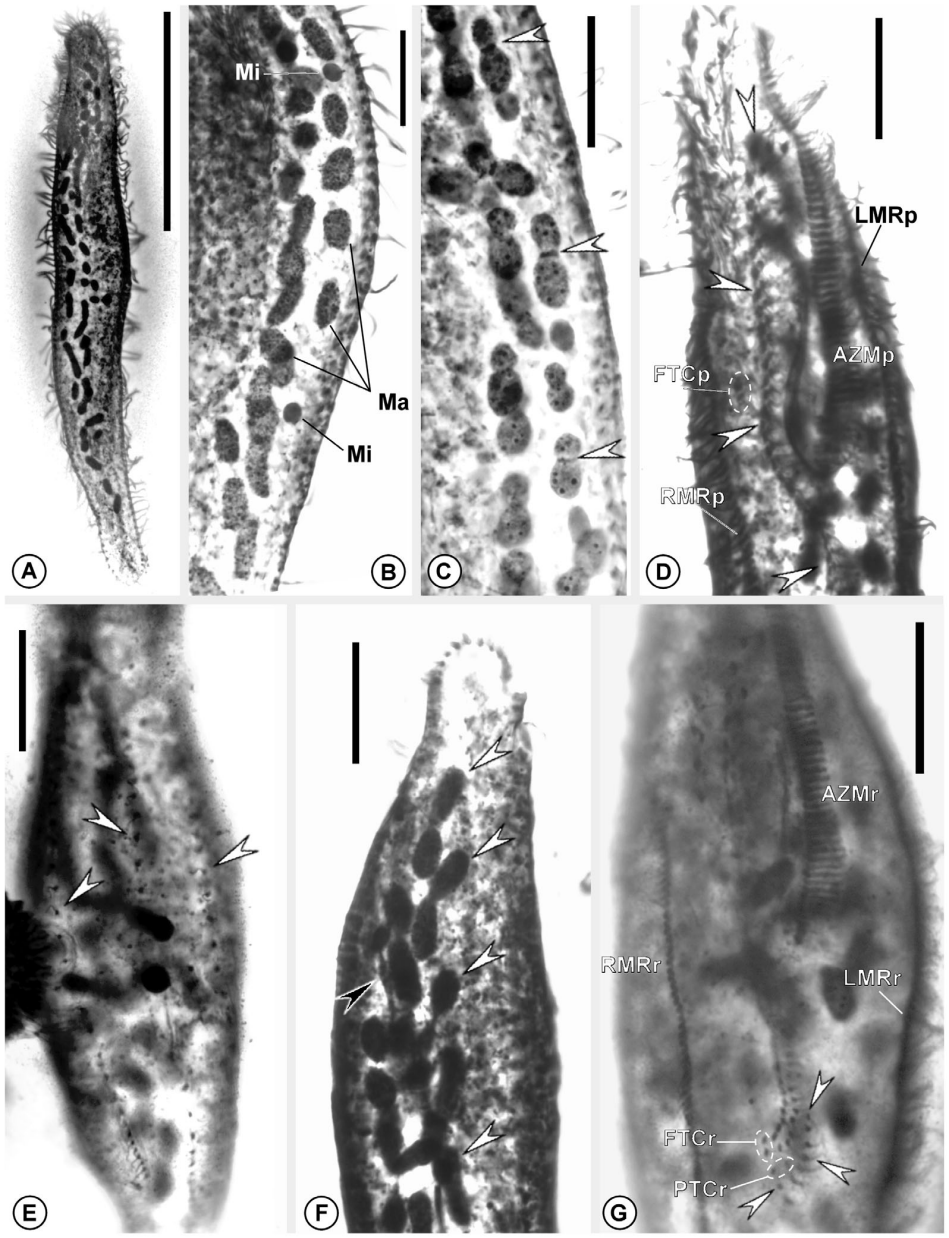
Optical micrographs of *Tetrakeronopsis silvanetoi* after protargol-impregnation. **A.** Holotype specimen viewed from dorsal side, showing the disposition of nuclear figure; **B.** Detail of nuclear apparatus shown from ventral side; **C.** Specimen showing macronuclear DNA replication bands (arrowheads); **D.** Proter of a late divider. Arrowheads show fronto-ventral set of juvenile cirri; **E.** Dorsal side of late divider opisthe showing juvenile dorsal kineties (arrowheads); **F.** Late divider showing morphogenesis of nuclear apparatus. Black arrowhead points to dividing micronucleus; white arrowheads show dividing macronuclei; **G.** Ventral side of reorganizer. Arrowheads indicate newly formed transverse cirri. Legend: AZMp – adoral zone (of membranelles) of proter; AZMr – adoral zone (of membranelles) of reorganizer; FTCp – frontoterminal cirri of proter; FTCr – frontoterminal cirri of reorganizer; LMRp – left marginal cirral row of proter; LMRr – left marginal row of reorganizer; Ma – macronuclear nodules; Mi – micronucleus; PTCr – pretransverse cirri of reorganizer; RMRp – right marginal cirral row of proter; RMRr – right marginal cirral row of reorganizer. Scale bars: **A.** 100 µm; **B–D**., **G**. 20 µm.

Nuclear apparatus formed by 30–47 ellipsoid macronuclear nodules measuring 5–13×2–6 µm, plus 3–8 more-or-less globular micronuclei measuring 4–6×3–5 µm; whole nuclear figure located mostly within left side of body and occupying almost entire body length (Figures 3A–B).

#### Morphogenesis

Only one very early divider, two late dividers, and some middle-stage reorganizers were present in the studied slides, from which some aspects of morphogenesis were unveiled (Figures 3C–G). The adoral zone of the proter is fully renewed and develops within the parental one. Gaps in adoral membranelles of lapel are likely formed in very late dividers or after cytokinesis. Ventral primordia of the proter develop without participation from cirri of rear corona; however, buccal cirrus very likely participates in the process. Midventral complex is formed as usual for pseudokeronopsid urostyloids, that is, from many short ventral primordia that each produces a cirral pair; the rightmost and second rightmost primordia each produces a pretransverse cirrus; transverse cirri are produced by the rightmost four to six primordia. The two frontoterminal cirri originate, as usual, from the rightmost ventral primordium. New marginal cirral rows and dorsal kineties originate from within parental structures. The parental ciliature is completely reabsorbed after cytokinesis. Macronuclear nodules become dumbbell shaped in very early dividers, each exhibiting a replication band. Unfortunately, it was not possible to determine if nodules fuse in a single mass prior to division or divide individually.

### Molecular Phylogeny of *Tetrakeronopsis silvanetoi* gen. nov., sp. nov

**Figure 4:**
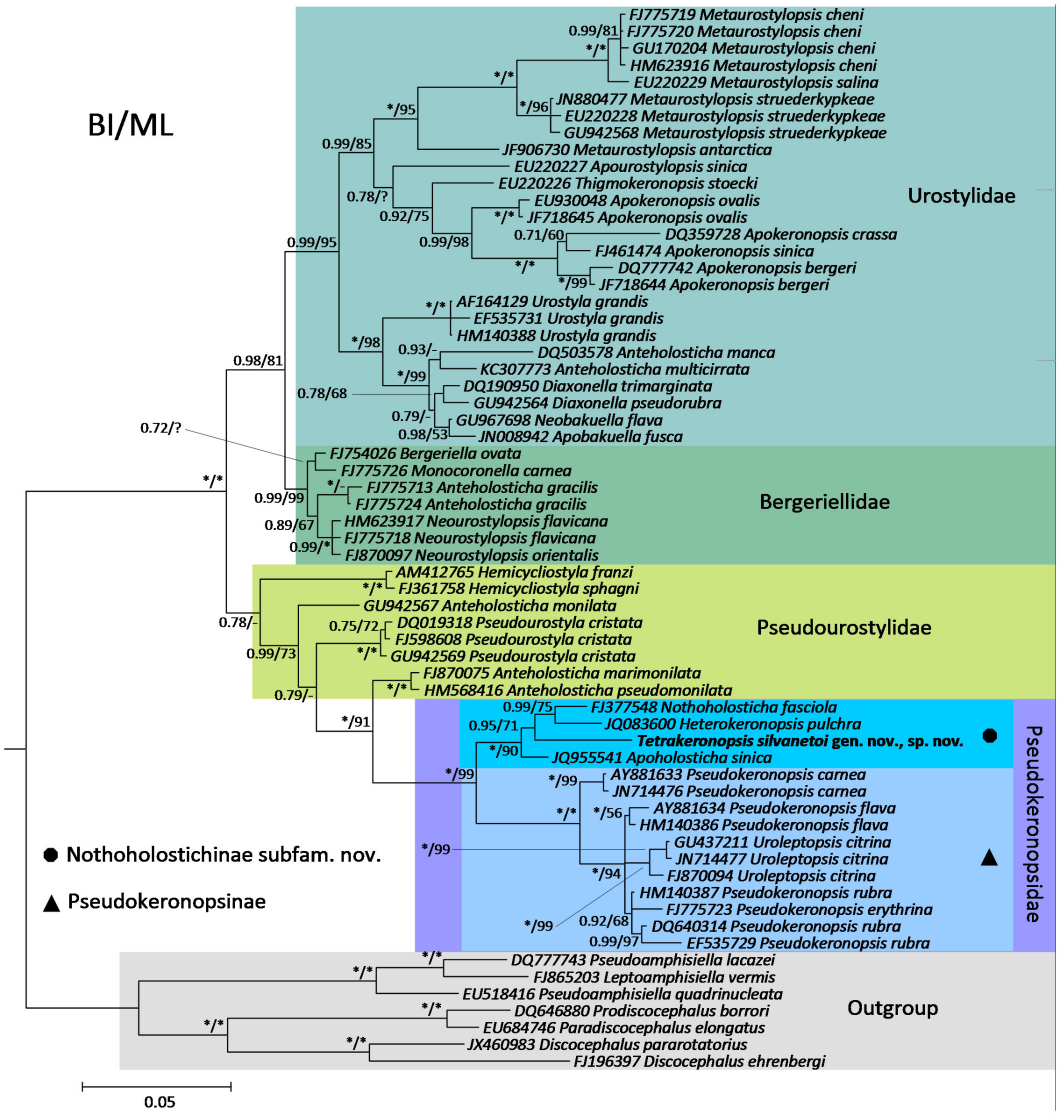
Bayesian inference (BI) phylogenetic tree of the core urostyloids showing the position of *Tetrakeronopsis silvanetoi* and the Nothoholostichinae. The numbers associated with nodes are Bayesian posterior probabilities and maximum-likelihood (ML) bootstrap values, respectively. NCBI/GenBank access codes are displayed left of species names. Legend: * – full support; - – support <50%; ? – cluster not hypothesized in the ML tree. Scale bar: five substitutions per 100 nucleotide positions.

The 18S rDNA fragment of *T. silvanetoi* was 1,456 nt long without gaps, and had a G+C content of 40.7 mol%. After alignment, it provided 1,637 positions of which 59.1% were identical to homologues found in the other sequences. The analyses recovered very similar phylogenetic patterns (Figure 4), differing only in the position of *Apourostylopsis sinica* (Shao *et al.*, 2008) Song et al., 2011, which diverged at the base of the *Metaurostylopsis* spp. cluster, and of *Monocoronella carnea* Chen *et al.*, 2011b, which was the sister group of *Anteholosticha gracilis* (Kahl, 1932) Berger, 2003, both in ML. The trees recovered three of the families commonly assigned with the core urostyloids, namely the Bergeriellidae Liu *et al.*, 2010, Pseudokeronopsidae Borror & Wicklow, 1983, and Urostylidae Bütschli, 1889. However, the Pseudourostylidae Jankowski, 1979 was paraphyletic in relation to the pseudokeronopsids. Moreover, the analyses also showed the genus *Anteholosticha* Berger, 2003 to be largely polyphyletic; hence, its representatives were classified according to their branching position (Figure 4).

**Figure 5:**
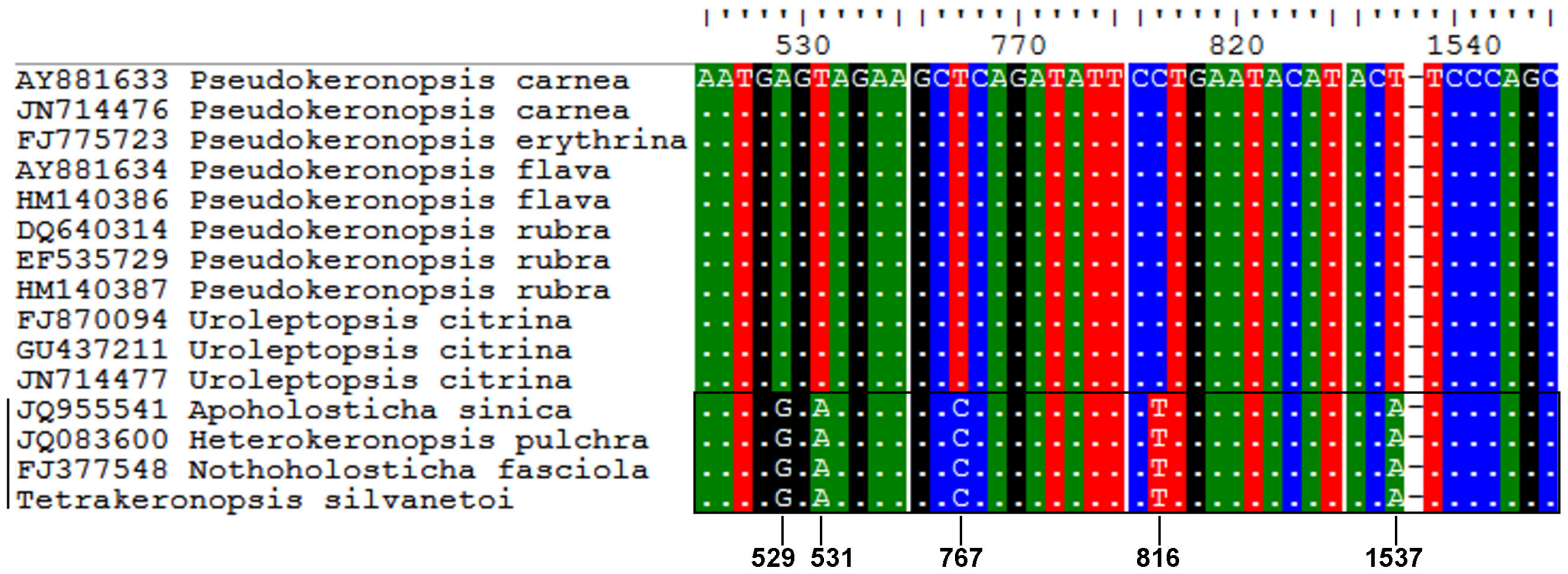
Part of the analyzed nucleotide matrix containing the Pseudokeronopsidae and showing the positions that are identity markers for the Nothoholostichinae subfam. nov. (rectangle) within the context of studied data. NCBI/GenBank accession codes are displayed left of species names.

*Tetrakeronopsis silvanetoi* consistently branched off the base of the *Heterokeronopsis pulchra* Pan *et al.*, 2013+ *Nothoholosticha fasciola* (Kahl, 1932) Li *et al.*, 2009 cluster and was preceded by *Apoholosticha sinica* Fan *et al.*, 2013. Those four terminals consisted of a strongly supported cluster which was an adelphotaxon of the *Pseudokeronopsis* spp.+*Uroleptopsis citrina* Kahl, 1932 group; thus, the Pseudokeronopsidae were always dichotomized, with one branch containing the species in which the anterior portion of the bicorona is formed by four frontal cirri, and the other branch containing those with a typical bicorona, namely the Nothoholostichinae and the Pseudokeronopsinae, respectively (Figure 4). Inspection of the nucleotide matrix revealed positions with putative molecular synapomorphies of the Nothoholostichinae that were unique within the analyzed sequences according to the 18S rRNA secondary structure: 529 G; 531 A; 767 C; 816 T; 1537 A, shown in Figure 5.

The *p*-distance between the 18S fragment of *T. silvanetoi* and that of *H. pulchra* was 0.026, while between *T. silvanetoi* and *N. fasciola*, but also *Apoholosticha sinica*, it was 0.025. Within-group mean *p*-distances were 0.022 and 0.014 for the Nothoholostichinae and Pseudokeronopsinae, respectively, and the mean *p*-distance between both clusters was 0.046 (Table 2).

## Discussion

### Comparison with Related Species

When compared to other pseudokeronopsids in which the anterior corona is formed by four frontal cirri, *T. silvanetoi* differs from both *Apoholosticha sepetibensis* (Wanick & Silva-Neto, 2004) Fan *et al.*, 2013, and *Apoholosticha sinica* in the number of frontoterminal cirri (two vs. four); the presence of buccal cirri (vs. absent); and the presence of distinct pretransverse cirri (vs. absent). Another difference rests on the number of transverse cirri. *Tetrakeronopsis silvanetoi* has 4–6 (on average 5), while *A. sepetibensis* has three and *A. sinica* has 2–4 transverse cirri. Moreover, *A. sepetibensis* has 4–6 dorsal kineties, while *T. silvanetoi*, like *A. sinica*, invariably exhibits three of such kineties [14], [23].

*Tetrakeronopsis silvanetoi* differs mostly from *Heterokeronopsis pulchra* in having two frontoterminal cirri located at their typical position (i.e. in the anterior region of body) [40]. According to Berger [2], [19], the frontoterminal cirri in hypotrichs correspond to the anteriormost cirri generated by the rightmost ventral primordium, which then migrate anteriad. In *H. pulchra*, those cirri are actually generated, however they do not migrate anteriad, remaining in a posterior midventral row [40]. Hence, frontoterminal cirri in *H. pulchra* are said to be absent because they do not occupy their typical position [2], [40]. Thus, *T. silvanetoi* differs from *H. pulchra* in lacking a midventral row formed by the rightmost ventral primordium (homologous to primordium VI in 18-FVT oxytrichids) (vs. present); and in the presence of pretransverse and transverse cirri (vs. absent). Additionally, when *T. silvanetoi* is compared to *H. pulchra*, the former shows more adoral membranelles (52–69 vs. 31–46), midventral cirral pairs (29–46 vs. 12–21), left marginal cirri (55–98 vs. 30–43), and right marginal cirri (54–90 vs. 35–52) than the latter [40].

In comparison with *Nothoholosticha fasciola, T. silvanetoi* differs in the presence of two frontoterminal cirri (vs. lacking in *N. fasciola*); however, unlike in *H. pulchra*, the cirri that are homologous to the frontoterminal ones are not generated in *N*. *fasciola* [41]. In their paper, Li *et al.* [41] transferred *Anteholosticha longissima* (Dragesco & Dragesco-Kernéis, 1986) Berger, 2006 to *Nothoholosticha* due to its supposed lack of frontoterminal cirri. According to Berger [2], the lack of such cirri in the original description by Dragesco & Dragesco-Kernéis [42] is possibly due to confusion with the right marginal row, which is indicated by the far anteriorly extending row [2]. Additionally, the frontal cirri are five in number and organized in a single corona, resembling *Monocoronella* Chen *et al.,* 2011b. Because of such, we transfer *Nothoholosticha longissima* to that genus as *M. longissima* comb. nov. This new combination is likely provisory since *M. longissima* apparently lacks buccal cirri, while all congeners display at least one [43]. It is worthy of note that a redescription of this organism, as suggested by Berger [2], is necessary to verify the absence of the buccal cirrus and elucidate other features, such as the dorsal ciliature pattern.

### Phylogeny

The phylogenetic trees obtained in the present study are generally in agreement with the literature concerning the internal kinships of the core urostyloids [20], [23], [24], [44], including their families and the paraphyly of pseudourostylids, which is recurrent in the literature [22], [23], [40]. In addition, the spreading of *Anteholosticha* spp. among various clusters also corroborates previous phylogenetic studies on urostyloids [23], [24], [26], [27], [45]. The heterogeneity of the genus *Anteholosticha* was already pointed out by Berger [2], [46]. Huang et al. [24] recently erected the genus *Arcuseries* Huang et al., 2014 for the cluster formed by *Anteholosticha petzi* Shao et al., 2011; *Anteholosticha scutellum* (Cohn, 1866) Berger, 2003 and “*Anteholosticha parawarreni*” (nomen nudum; see [24]), which consistently groups outside the core urostyloids in molecular trees [24], [26]. Hence, *Anteholosticha* will likely be split into more genera in the future.

Moreover, the data supporting the phylogenetic patterns herein recovered, as expressed by posterior probabilities and bootstrap values, is also consistent with the literature, since the 18S core urostyloid clusters are hypothesized mostly with strong data support among the hypotrichs phylogeny [21], [23].

The dichotomy separating the pseudokeronopsids in which the anterior corona is formed by four frontal cirri and those with a typical bicorona has been progressively shown in the literature, as further representatives of the former were discovered and subjected to phylogenetic analyses [20], [23], [40], [41]. The inclusion of *T. silvanetoi* further corroborated this emerging pattern, consequently leading to the naming of a natural group, the Nothoholostichinae.

### Foundation for the Erection of Nothoholostichinae Subfam. nov. and *Tetrakeronopsis* gen. nov

In their paper, Borror & Wicklow [47] included the Thigmokeronopsinae Wicklow, 1981 in the Pseudokeronopsidae, thus subdividing it into two subfamilies. Molecular data has suggested the former to be a monophyletic taxon that belongs to the Urostylidae instead of Pseudokeronopsidae, as recently proposed by Huang et al. [24]. Based on molecular phylogenetic analyses [20], [23], [24], [40], the Pseudokeronopsidae were consistently split into two natural groups – the Pseudokeronopsinae Borror & Wicklow, 1983 and the newly erected Nothoholostichinae. The phylogenetic pattern recovered in the present paper suggests the typical bicorona of the Pseudokeronopsinae to be a plesiomorphic feature, inherited from a pseudourostylid-like last common ancestor. Hence, the peculiar composition of the anterior corona in *Apoholosticha*, *Heterokeronopsis*, *Nothoholosticha* and *Tetrakeronopsis* is herein regarded as a putative synapomorphy of the Nothoholostichinae within the Pseudokeronopsidae, and a feature that likely evolved by a reduction in the number of frontal primordia. Curiously, the morphometric data from *Heterokeronopsis pulchra*, shown in [40], indicate that occasional specimens may have five frontal cirri. Hence, some slight variations in the four-cirri pattern are expected to occur in the atypical bicorona of Nothoholostichinae.

Additional features which may be of taxonomic relevance for diagnosing the Nothoholostichinae are the presence of a longitudinal groove left of midventral complex and the split of adoral zone by a small gap (which also occurs in *Uroleptopsis* Kahl, 1932). Contractile vacuoles, when present, are generally located behind the equatorial level of body, and cytoplasm contains numerous inclusion bodies, which are also present in the Pseudokeronopsinae [2], [14], [23], [40], [41]. Moreover, as mentioned in the above section, specific conserved positions that may provide a molecular identity for the Nothoholostichinae among the core urostyloids were found in our alignment (Figure 5), thus strengthening the establishment of this new subfamily.

Lastly, the new genus *Tetrakeronopsis* is established based on a unique combination of morphologic features for a nothoholostichine pseudokeronopsid (Table 3), namely, the presence of two frontoterminal cirri; presence of buccal, transverse and distinct pretransverse cirri; and lack of a posterior midventral cirral row.
